# Supplementary material for: Scalable magnetoreceptive e-skin for energy-efficient high-resolution interaction towards undisturbed extended reality
Source: Nat Commun. 2025 Feb 14;16:1647. doi: 10.1038/s41467-025-56805-x (PMC11828903; doi:10.1038/s41467-025-56805-x)
Supplement: Supplementary file 1 — Supplementary Information [file 41467_2025_56805_MOESM1_ESM.pdf]

Supplementary Information

Scalable magnetoreceptive e-skin for energy-efficient high-resolution interaction towards undisturbed extended reality

Authors:

Pavlo Makushko[1#], Jin Ge[1#*], Gilbert Santiago Cañón Bermúdez[1], Oleksii Volkov[1], Yevhen Zabila[1], Stanislav Avdoshenko[2], Rico Illing[1], Leonid Ionov[3], Martin Kaltenbrunner[4,5], Jürgen Fassbender[1], Rui Xu[1*], Denys Makarov[1*]

Affiliations:

[1] Helmholtz-Zentrum Dresden-Rossendorf e.V., Institute of Ion Beam Physics and Materials Research, Bautzner Landstrasse 400, 01328 Dresden, Germany

[2] Institute for Solid State Research, Leibniz Institute for Solid State and Materials Research Dresden, 01069 Dresden, Germany

[3] Faculty of Engineering Science, Biofabrication, University of Bayreuth, Ludwig-Thoma-Str. 36a, 95447 Bayreuth, Germany

[4] Division of Soft Matter Physics, Institute for Experimental Physics, Johannes Kepler University, Altenberger Str. 69, 4040 Linz, Austria

[5] Soft Materials Lab, Linz Institute of Technology, Johannes Kepler University, Altenberger Str. 69, 4040 Linz, Austria

Correspondence and requests for materials should be addressed to J.G., R.X. and D.M. (email: gejin@mail.sysu.edu.cn, r.xu@hzdr.de, d.makarov@hzdr.de)

# These authors contributed equally to this work

Supplementary Figures

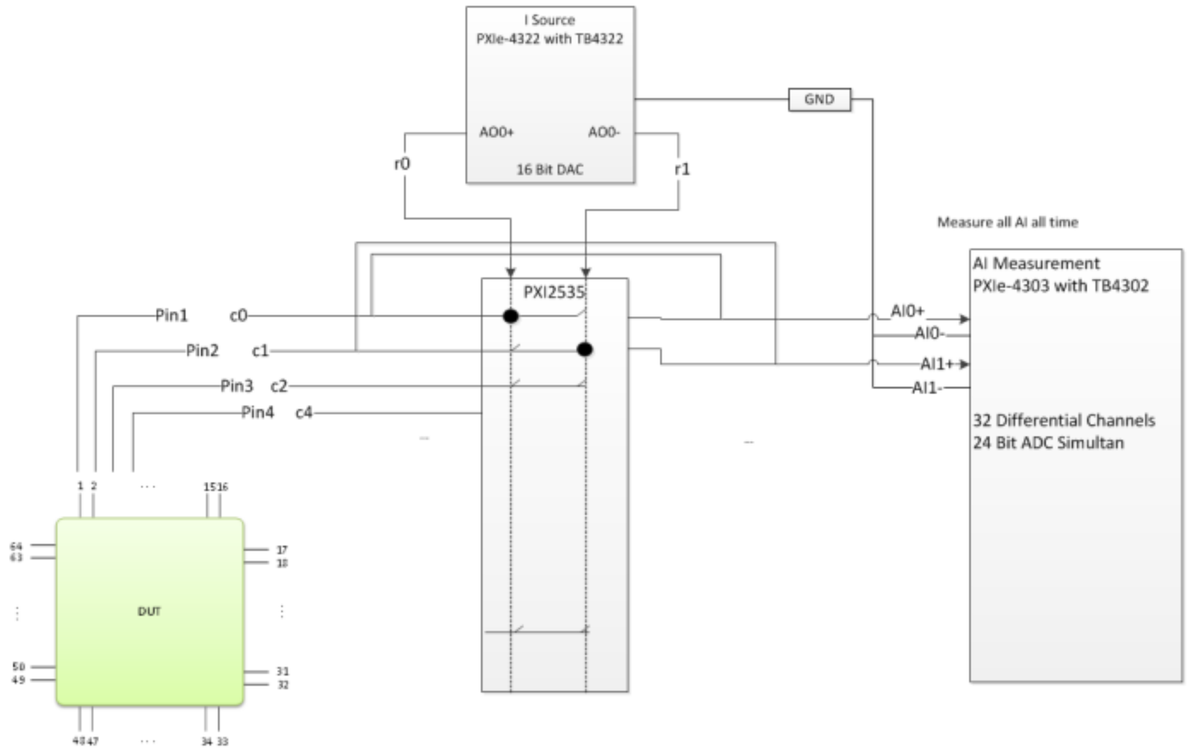

Supplementary Figure 1. Connection scheme for the EMRT readout system. A matrix switch (PXI 2535) routes the driving current and voltage measuring pins from the analog input module (PXIe-4303) to the device under test (DUT), in this case the GMR mesh sensor. The voltage measurement pins are switched sequentially along the perimeter of the DUT until completing one round. Next, the current driving pins are switched one position along the perimeter and the measurement starts over. This procedure is repeated until all possible combinations of current and voltage pins have been acquired and a full matrix of voltages (Supplementary Table S1) is completed.

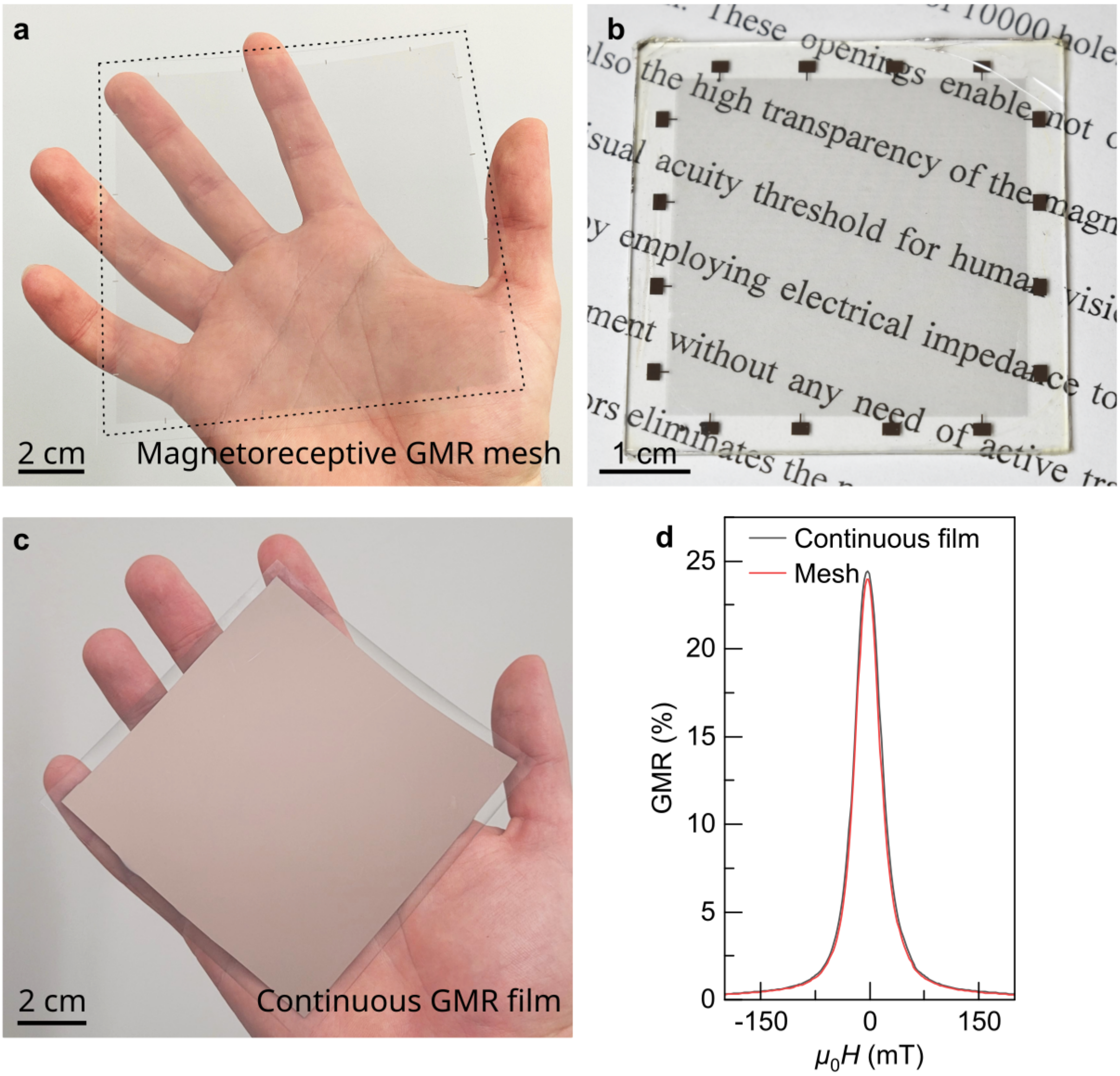

Supplementary Figure 2. Transparent magnetoreceptive GMR mesh. (a) Photograph of the 120x120 mm^2 GMR mesh sensor prepared on a PET polymeric substrate. (b) 40x40 mm^2 GMR mesh sensor prepared on mylar foil (3 μm-thick). For ease of lithographic processing the sensor is adhered to a glass piece. (c) Photograph of the continuous GMR film (size of 100x100 mm^2) on PET substrate. (d) Typical magnetoresistive response of the continuous and mesh-patterned GMR sensors.

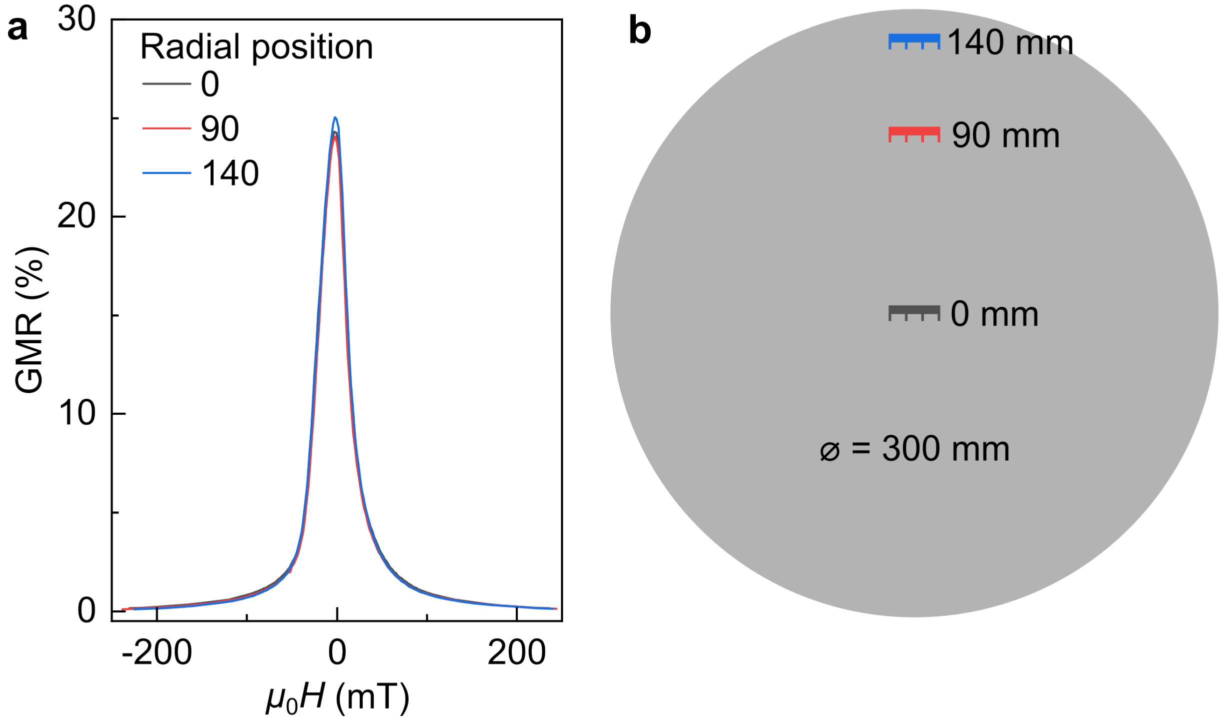

Supplementary Figure 3. Spatial homogeneity of the Co/Cu GMR multilayers. (a) Magnetoresistive response of the GMR layer deposited onto a 300-mm-diameter silicon wafer. The legend corresponds to the radial position from the center of the wafer, as depicted in panel (b). The GMR performance is homogeneous throughout the whole surface, while a slight increase in the resistance (about 5%) is observed at the very edge. Sample resistance measured at zero magnetic field in the center of the wafer (position indicated as “0”) is 5.19 Ohm. Sample resistance measured at the location, which is 90 mm away from the center of the wafer (position indicated as “90”), is 5.15 Ohm. Sample resistance measured at the edge of the sample, which is 140 mm away from the center of the wafer (position indicated as “140”), is 5.40 Ohm.

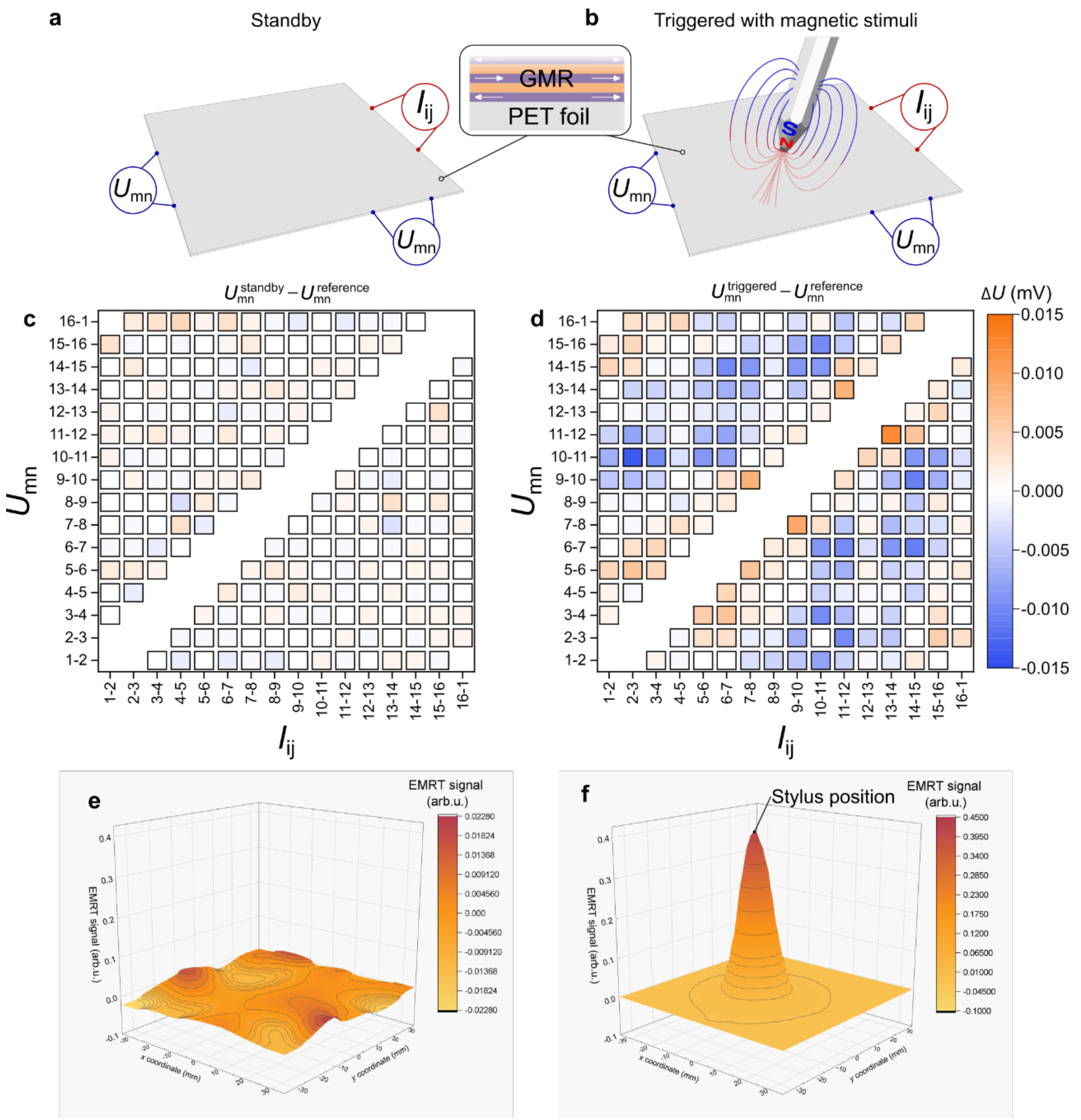

Supplementary Figure 4. Principle of the EMRT data acquisition using adjascent-adjscent measurement protocol. (a) sketch of GMR-based extended magneto-receptor electrically connected along its perimeter. (b) Heatmap of voltage differences (U_{actual} - $U_{reference}$) recorded on the magnetoreceptor in standby mode (no magnetic input). The measured voltage difference do not exceed 2 μV, which is on the noise level. (c) Corresponding EMRT signal map showing no sizeable variation in the resistivity distribution. (d) sketch of the GMR-based extended magnetoreceptor triggered with magnetic stylus (lines represent localized magnetic stray field lines outgoing from the stylus tip). (e) Heatmap of voltage differences (U_{actual} - $U_{reference}$) recorded when magnetoreceptor is magnetically triggered in the center. GMR-induced electrical response reaches 15 μV. (f) Corresponding EMRT signal map shows change in the resistivity of the magnetoreceptor in its portion exposed to the magnetic stylus stray fields.

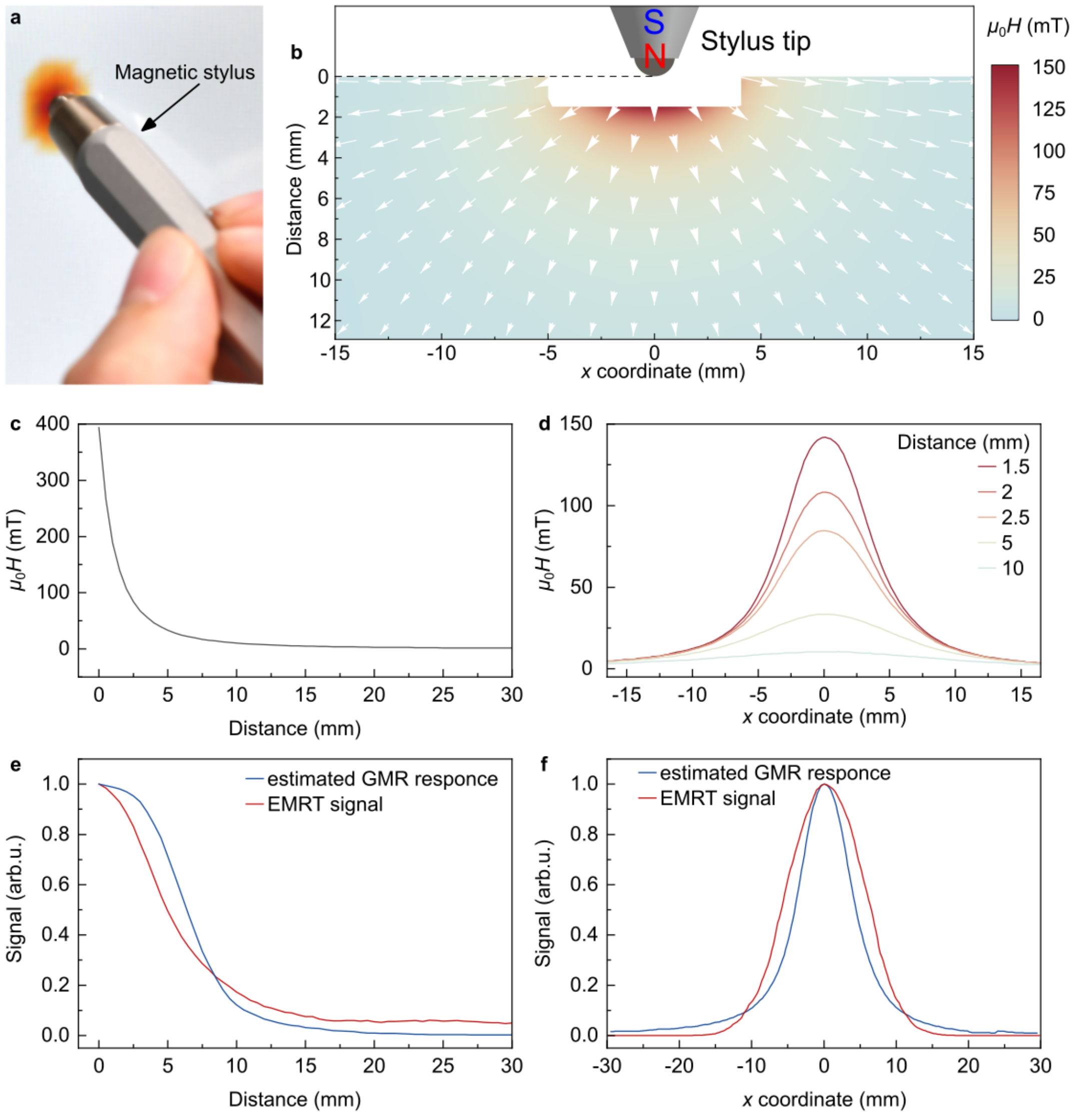

Supplementary Figure 5. Magnetic stylus characterization. (a) A photograph of the magnetic stylus used for the interaction with the EMRT-based imperceptible GMR mesh. (b) Map of the stray field around the magnetic stylus tip. Color fill represents the magnitude of magnetic flux $\mu_0 H$ and arrows show the magnetic vector direction. (c) Line profile of the stray field map on panel (b) as function of the distance from the tip of magnetic stylus. Magnetic flux rapidly decreases with distance: from 400 mT on the magnet surface down to 30 mT at the 5 mm distance. (d) Line profiles of the magnetic field map along the *x*-coordinate at different distance from the stylus tip. (e) Variation of the EMRT detected magnetoresistive response magnitude with the distance between the magnetic stylus and the sensor mesh plane and the estimation of the GMR response obtained by multiplying the magnetic flux magnitude (panel (c)) and the typical GMR response of the sensor mesh (Supplementary Figure 2 and Figure 1i in Main text). Starting from the distance of 15 mm the magnetic signal is below the noise level of the EMRT setup and does not allow to reliably reconstruct the position of magnetic stylus (See also Figure 2f-g of the main text). (f) Line profile (along *x* coordinate) of the measured and expected EMRT signal at stylus distance from the sensor of 2mm. Estimation obtained by multiplication of the magnetic flux distribution from panel (d) and the typical GMR response of the sensor mesh.

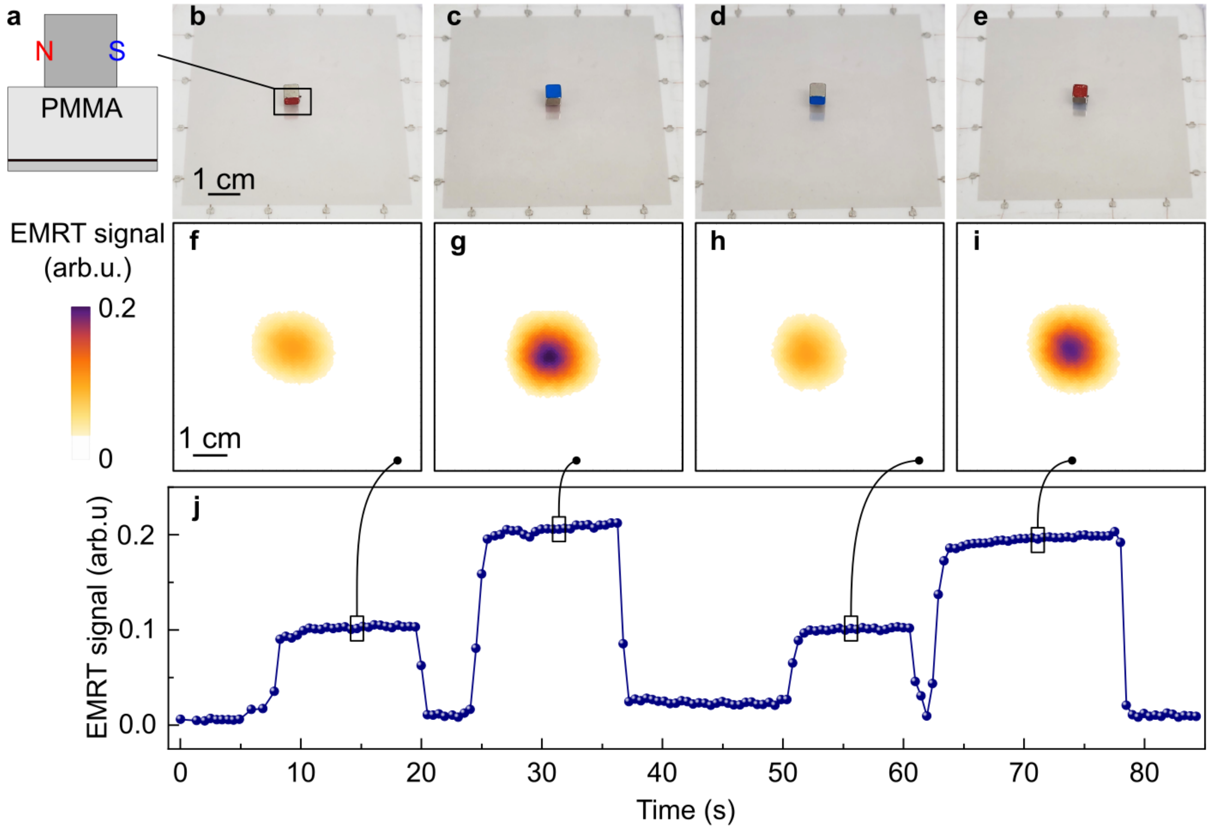

Supplementary Figure 6. Sensitivity of the EMRT conditioned magnetoreceptor to the magnet orientation. (a) Schematics of the experiment: a cube-shaped permanent magnet with marked poles (North – red and South – blue) is placed above the large-area magnetoreceptor. A 5-mm-thick PMMA sheet is used as a spacer between the magnet and the magnetoreceptor. (b-e) Photographs of the bar magnet placed above the magnetoreceptor with different orientation of its poles and (f-i) the corresponding reconstructed resistance maps. All panels are plotted using the same color scaling. (j) The EMRT signal timeline recorded during the experiment. The EMRT reconstructed GMR response of the magnetoreceptor is twice smaller when the magnet is placed on a side, as compared to when the magnet is facing the pole towards the magnetoreceptor plane. This is related to the higher magnetic flux close to the poles of the magnet compared to its sides.

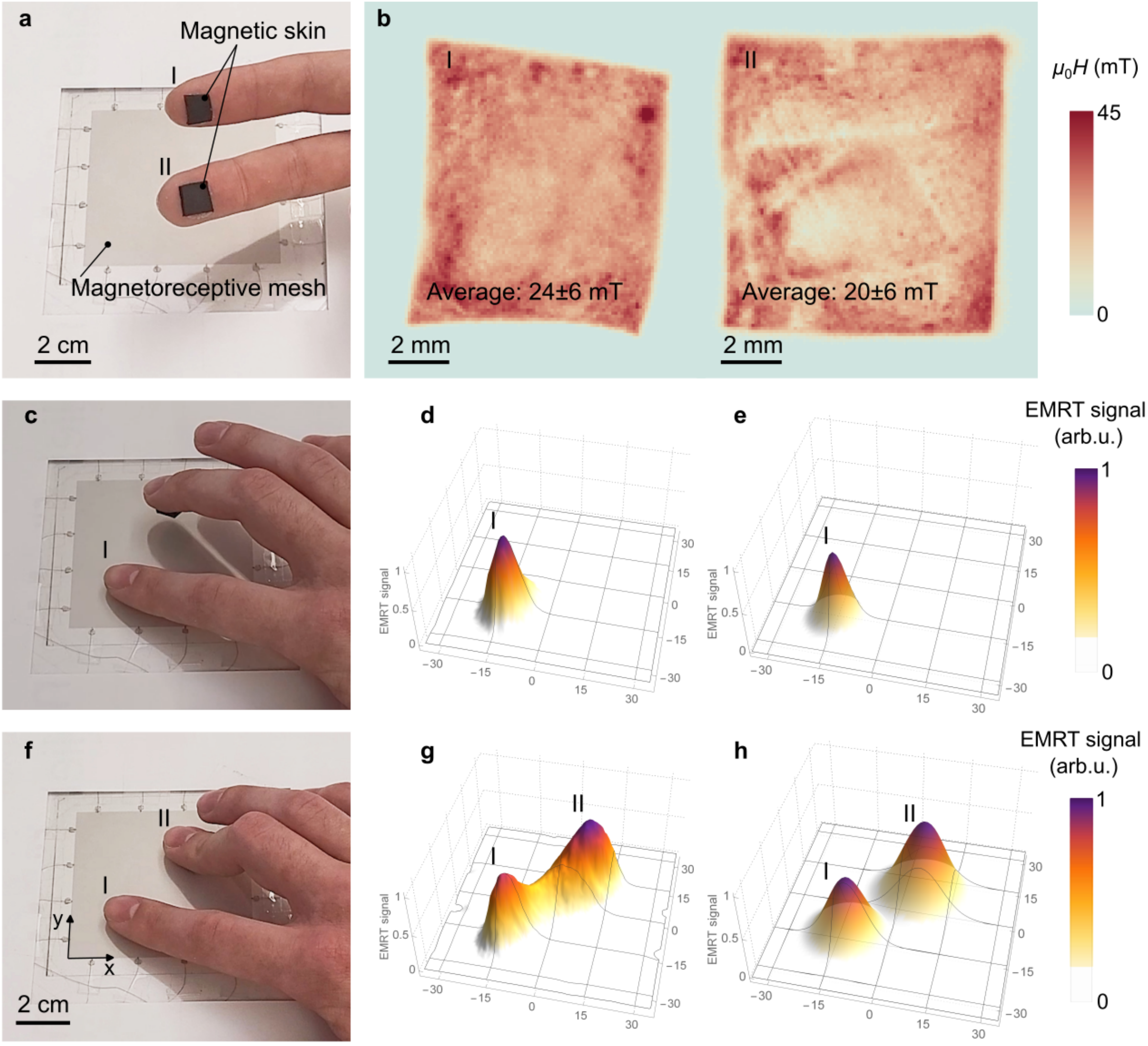

Supplementary Figure 7. Two-point interaction with the EMRT conditioned large-area magnetoreceptor. (a) A selected frame from the Supplementary Movie 6 showing two mechanically flexible magnetic skins (labelled as "I" and "II") attached to fingertips of a user. (b) Images of the magnetic stray field profiles measured at the magnetic skin surface. The measurement of stray fields is carried out using cmos-magview device (matesy GmbH, Germany). (c,f) Selected frames of the Supplementary Movie 6 showing a single-point and two-point interactions with the magnetoreceptive mesh using magnetic skin on fingertips. (d,g) The corresponding EMRT reconstruction of the magnetoresistive response and (e,h) double Gaussian fit analysis of the data allowing to locate the position of each magnet. In panel (e) only one Gaussian peak is shown as the second one is of low intensity (fingertip with the magnetic skin II is lifted away from the sensor surface).

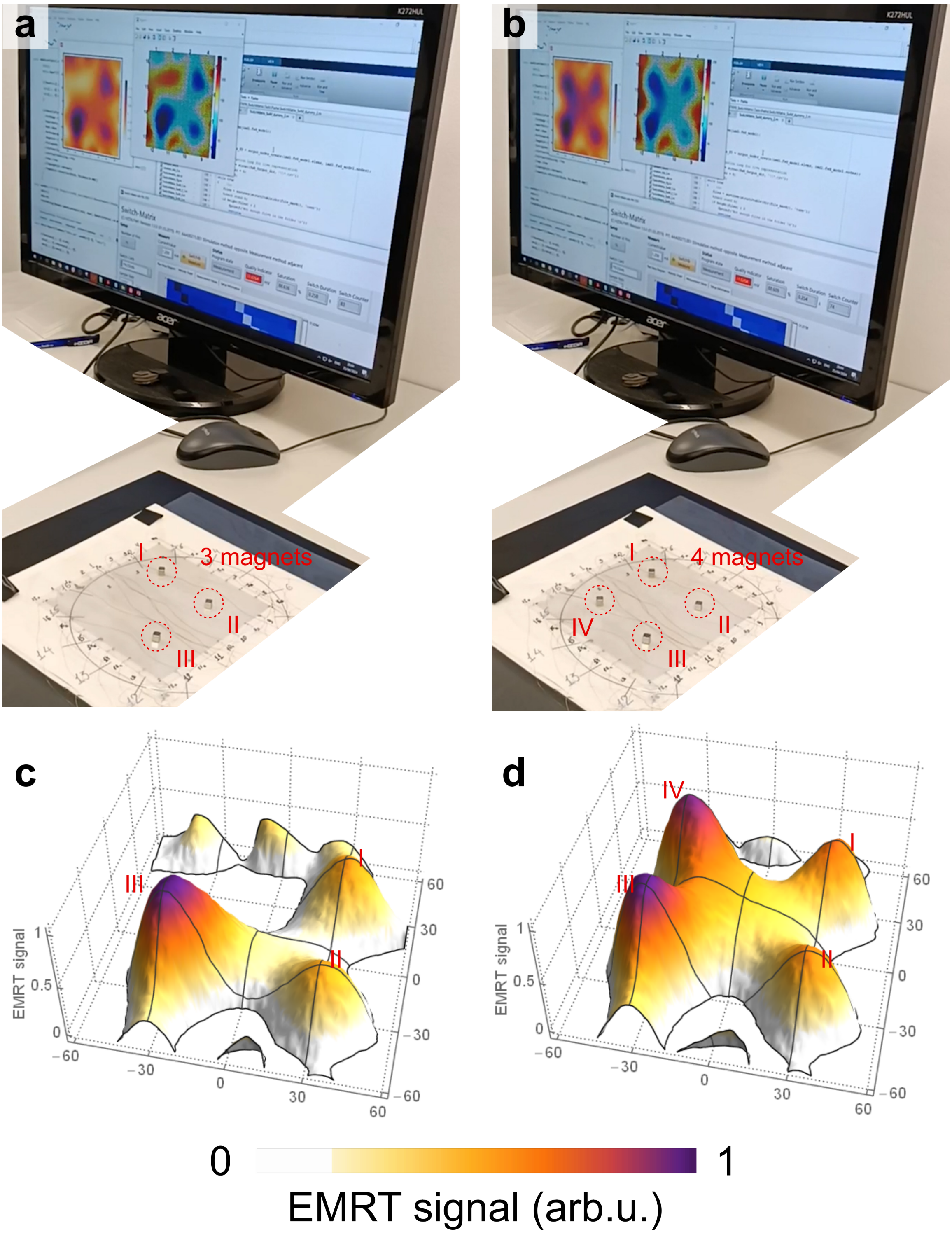

Supplementary Figure 8. Identifying multiple magnetic inputs. (a,b) Selected frames of the Supplementary Movie 7 showing the experimental setup. A large-area magnetoreceptor is placed above the steel plate and is used to locate multiple cube-shaped permanent magnets. The corresponding EMRT reconstructed magneto-resistive response is shown on a computer screen and in panels (c,d). The positions of four magnets are reconstructed as peaks on the resistance maps. The magnetoreceptor was capped with 125-μm-thick PET foil to prevent shortcutting onto the metallic surface of the magnets.

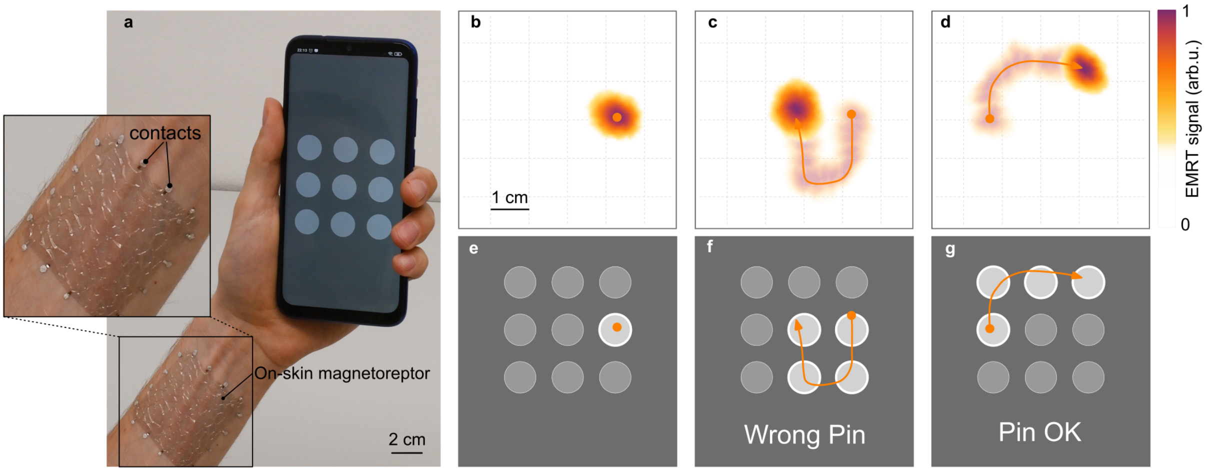

Supplementary Figure 9. Magnetoreceptive permeable GMR mesh for on-skin applications. (a) A frame from the Supplementary Movie 1 showing magnetoreceptive e-skin applied onto a wrist of a person and used for interfacing with a smartphone. An inset shows the magnified image of the magnetoreceptor and conductive paint contact points. The interaction with e-skin is realized relying on the magnetosensitive skin, the recognized input is transferred to the smartphone. (b-d) A series of reconstructed EMRT interactive segments shown in the Supplementary Movie 1, suggesting touchless input of incorrect and correct graphical PIN codes to unlock the smartphone. The recorded and interpolated stylus trajectory is shown with a solid orange line. (e-g) Representation of the corresponding snapshots of the smartphone interface.

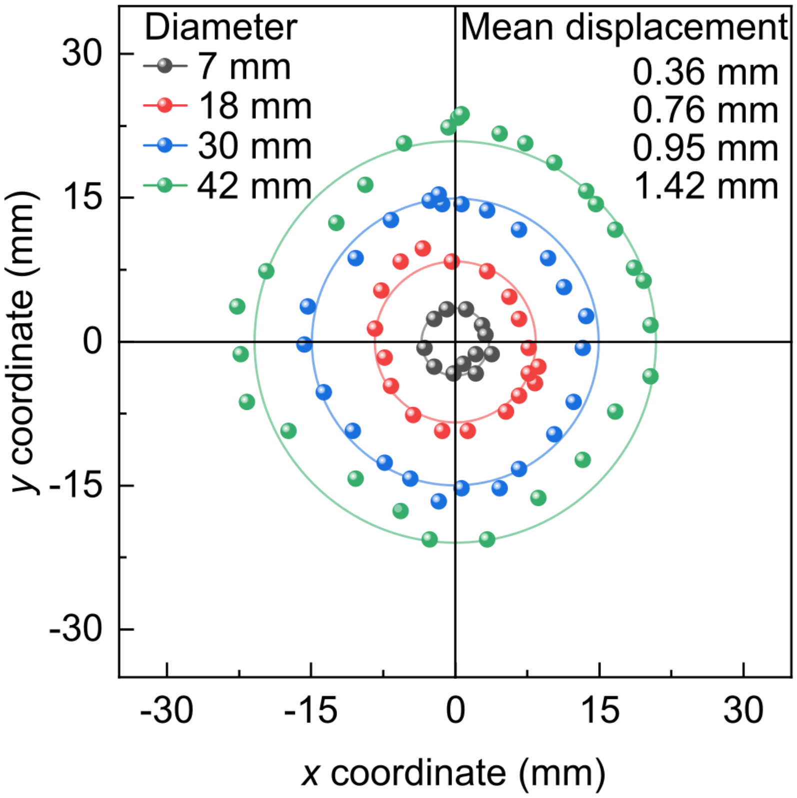

Supplementary Figure 10. A set of concentric circles hand-drawn using the EMRT based touchless interaction setup. The mesh size is 70 x 70 mm^2, EMRT is exploited in 16-contacts probe geometry and 1 mA probing current. Lines represent the intend-to-draw circles and symbols stand for experimental result. A circle of a 7 mm diameter is reliably drawn. The average displacement between the recorded and intended trajectory is within a limit of 1 mm. A larger average displacement of 1.4 mm at the largest drawn circle is related to the human factor, as the shapes were drawn by hand. The result indicates high spatial resolution of the EMRT method below 1 mm.

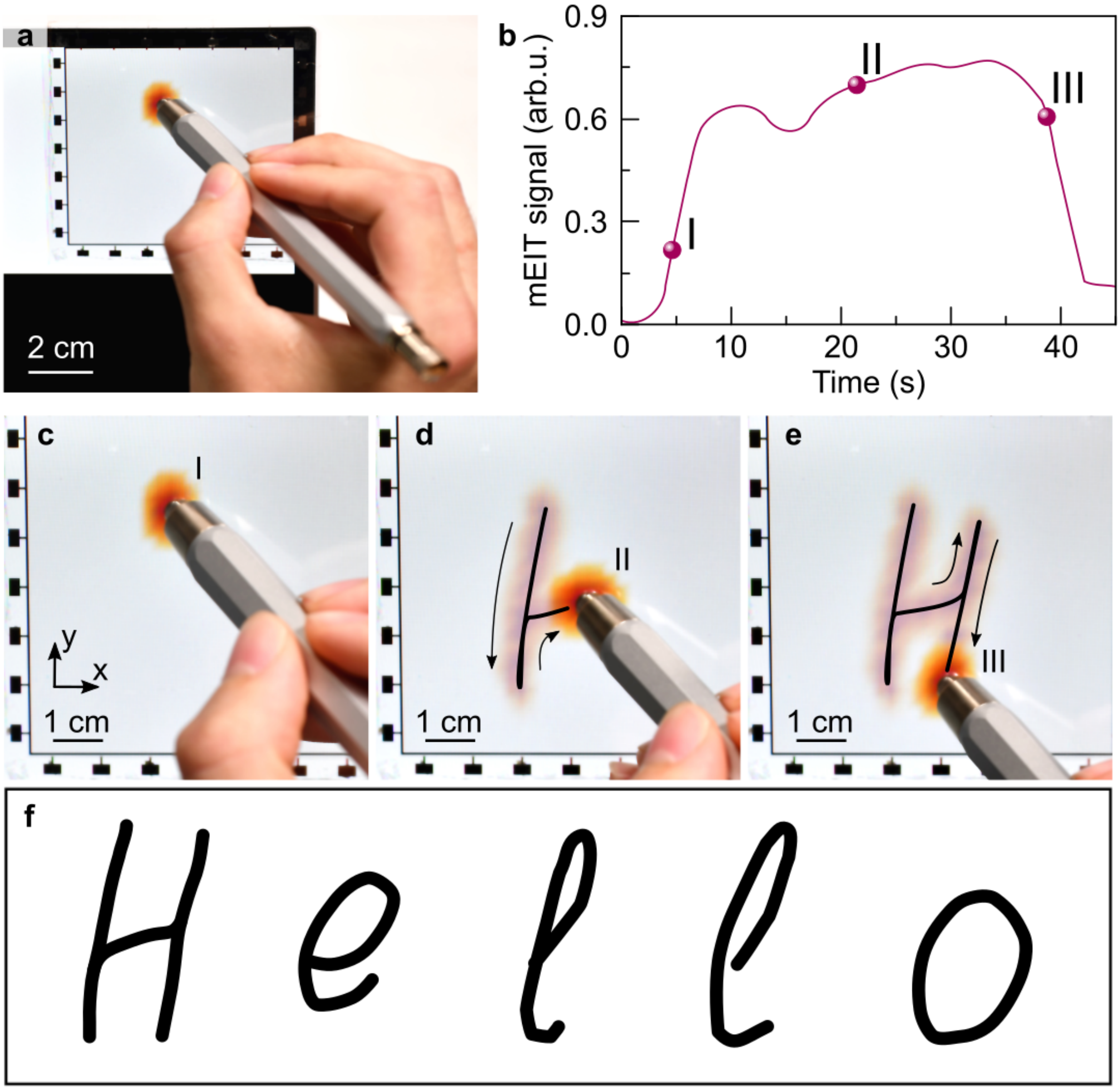

Supplementary Figure 11. Transparent magnetoreceptive mesh sensor for large-area magnetic field mapping. (a) Transparent GMR mesh sensor (size: 70x70 mm^2) located in a corner of a monitor and its resistance state is monitored using EMRT algorithm relying on 16 contact geometry. An orange spot on the background corresponds to localized drop of the resistance of the mesh due to GMR effect. Magnetic stylus is used as an input device to insert a handwritten word "Hello", see also Supplementary Movie 2 and Figure 2 of the main text. (b) The amplitude of the input signal during the input of handwritten character "H". (c-e) A sequence of representative frames of Supplementary Movie 2 depicting the EMRT-based touchless writing process of the character "H". A bright orange spot indicates the local drop of the resistance reconstructed with EMRT algorithm and shallowed orange trace represent the trajectory of magnetic stylus hovering above the mesh sensor. (f) A word "Hello" written using transparent magnetoreceptive sensor, the full process is shown in Supplementary Movie 2.

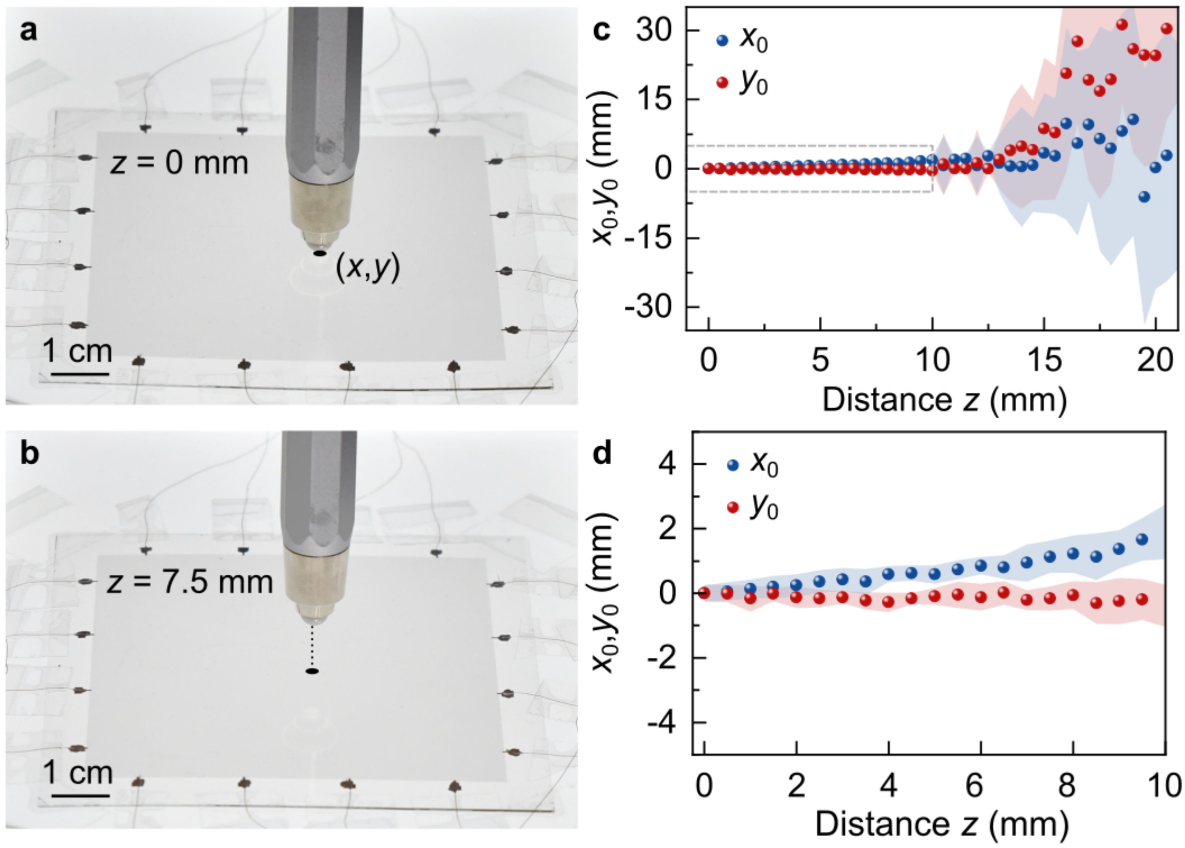

Supplementary Figure 12. Accuracy of the spatial acuity of the EMRT conditioned magnetoreceptor. (a) Magnetic stylus is positioned above a large-area magnetoreceptor at a fixed (*x*,*y*) position and is vertically moved away (b) from the sensor plane (along z-direction). (c) The deviation of the EMRT reconstructed stylus position from the (*x*,*y*) with the increasing distance along z-axis. (d) A zoomed in region of the panel (c) showing the deviation of the reconstructed position from the actual position of being less than 1 mm as the distance from the sensor plane is less than 10 mm. In panels (c) and (d) symbols represent the mean deviation of the reconstructed position over the 30 EMRT recorded frames and the colored background stands for the standard deviation.

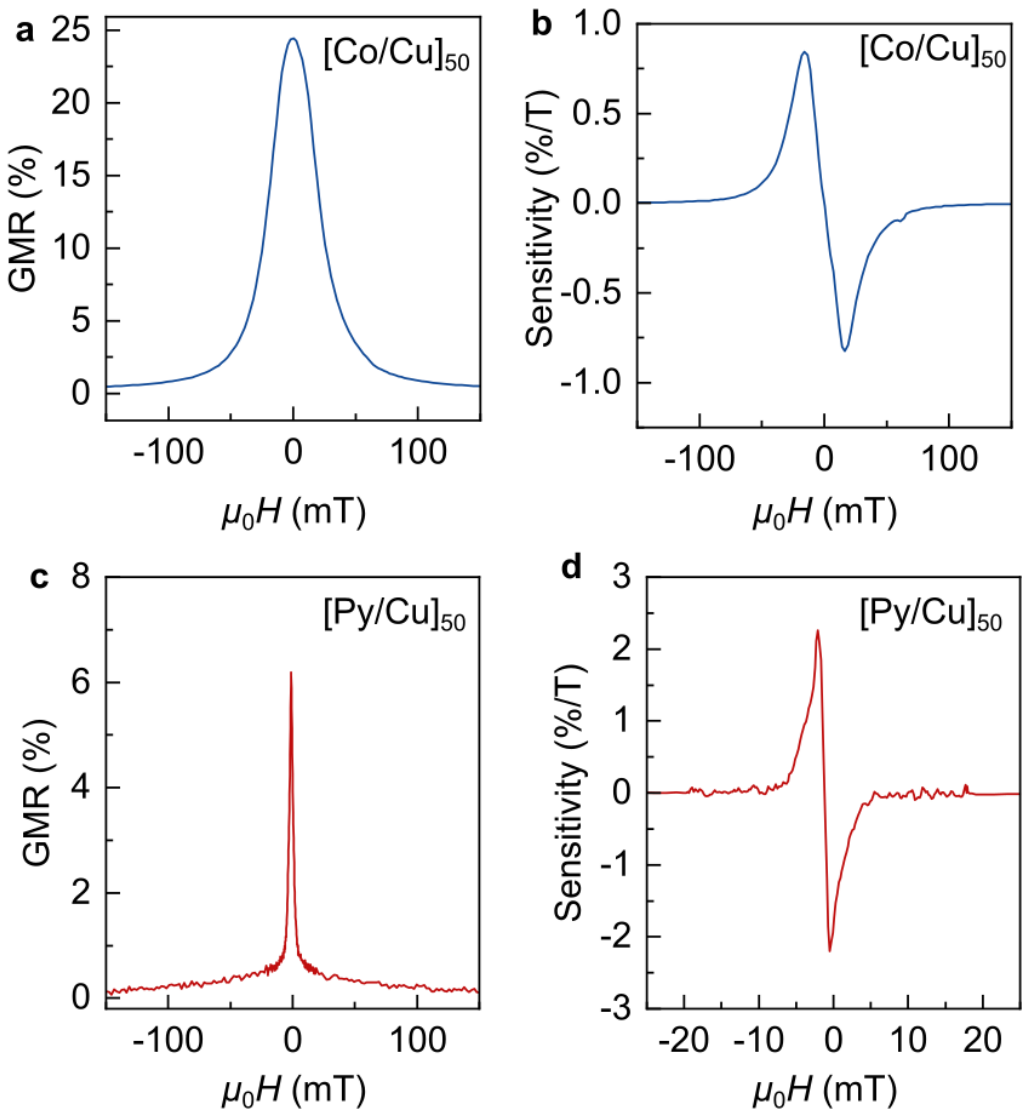

Supplementary Figure 13. Magnetoresistive performance of $[Cu/Cu]_{50}$ and $[Py/Cu]_{50}$ multilayer stacks. Typical magnetoresistive curves (a,c) and their first derivatives (b,d) that represent performance characteristics of magnetoreceptive elements. The Co/Cu multilayers reveal higher GMR ratio of 25 % compared to Py/Cu multilayer (with a typical GMR magnitude of about 7 %). However, the Py/Cu sensors reveal higher sensitivity, reaching 2%/T and are characterized by a smaller saturation field. These differences arise from the stronger magnetic anisotropy and stronger electron scattering of cobalt, as well as the soft magnetic properties and weak electron scattering behavior of permalloy. From the application point of view, this information gives a clue that the composition of the GMR layers (Py-based or Co-based) should be optimized for the specific needs. Permalloy-based magnetoresistive elements are more suitable for measuring weak magnetic signals, while cobalt-based sensors may be used in more general scenarios requiring a larger magnetic field range.

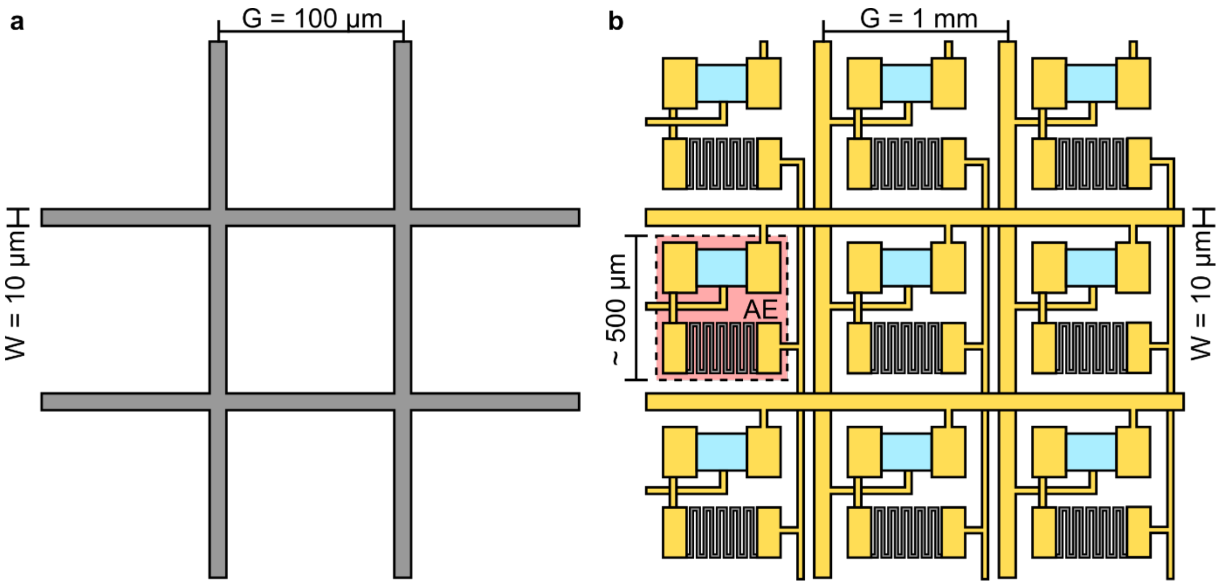

Supplementary Figure 14. Comparison between the filling factors of a magnetosensitive mesh (a) and the approximate configuration of a standard active transistor matrix seen in other works[1,2] (b). The parameters G, W and AE represent the mesh spacing, trace width and active element (sensor + transistor) size, respectively. The filling factor of the mesh sensor with 10 µm linewidth and 100 µm pitch is as small as 19 %.

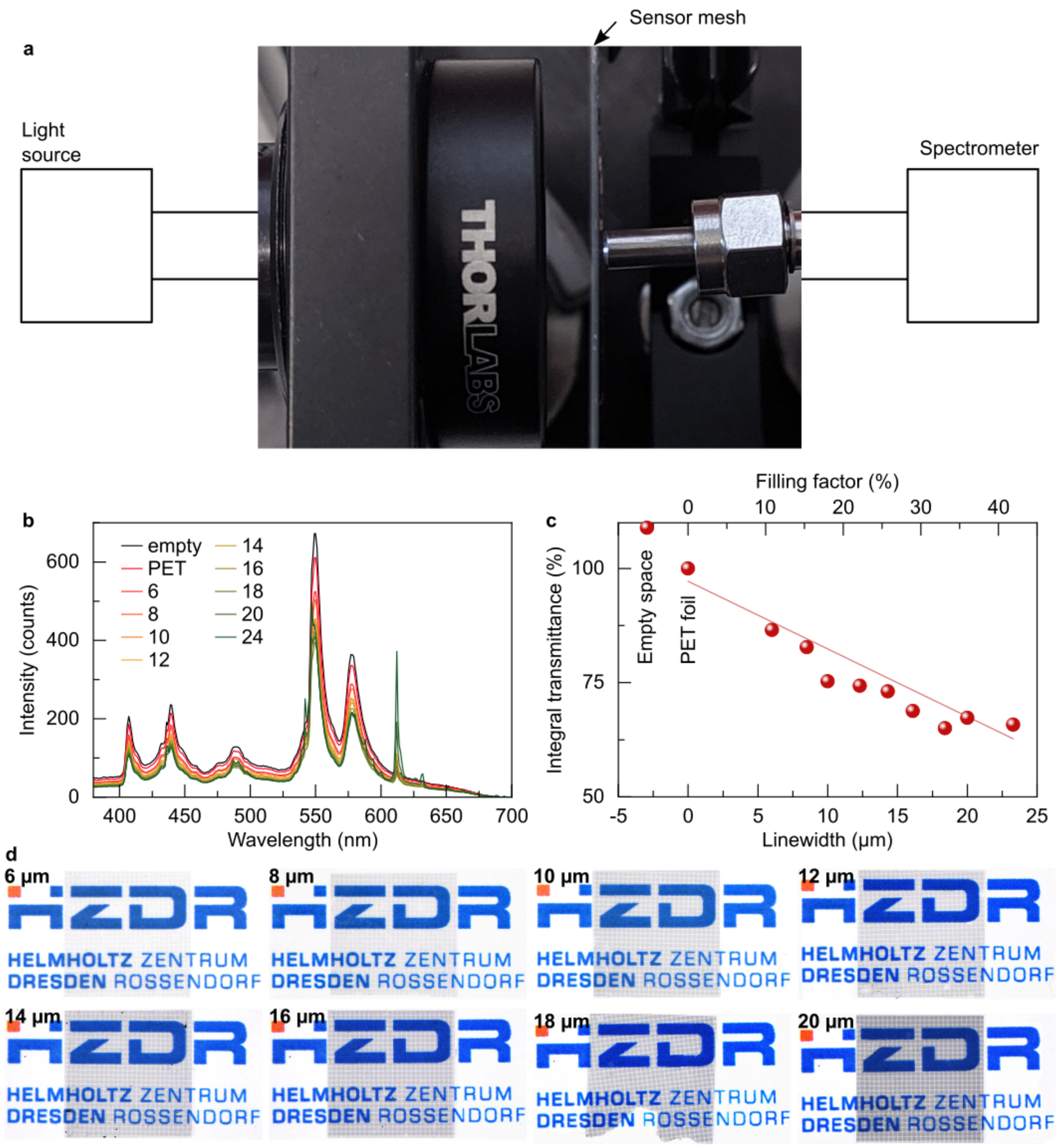

Supplementary Figure 15. Optical transparency of GMR mesh on PET polymer substrate. (a) Schematic of the measurement setup. White light from the light source is directed onto the transparent mesh sensor providing a spot size of about 1 cm. Transmitted light is collected and analysed using a digital spectrometer. (b) Measured transmitted light spectra for a series of meshes with 100 µm pitch and linewidth ranging from 6 to 24 µm. The filling factor is varied from 10 % to 40 %. (c) Calculated values of integral light transmittance (shown also in Figure 2c of main text). A clean PET substrate was taken as reference, optical transmittance of the PET substrate itself was estimated to be about 92%. (d) A series of images of transparent e-skins with different linewidth placed oved the HZDR logo.

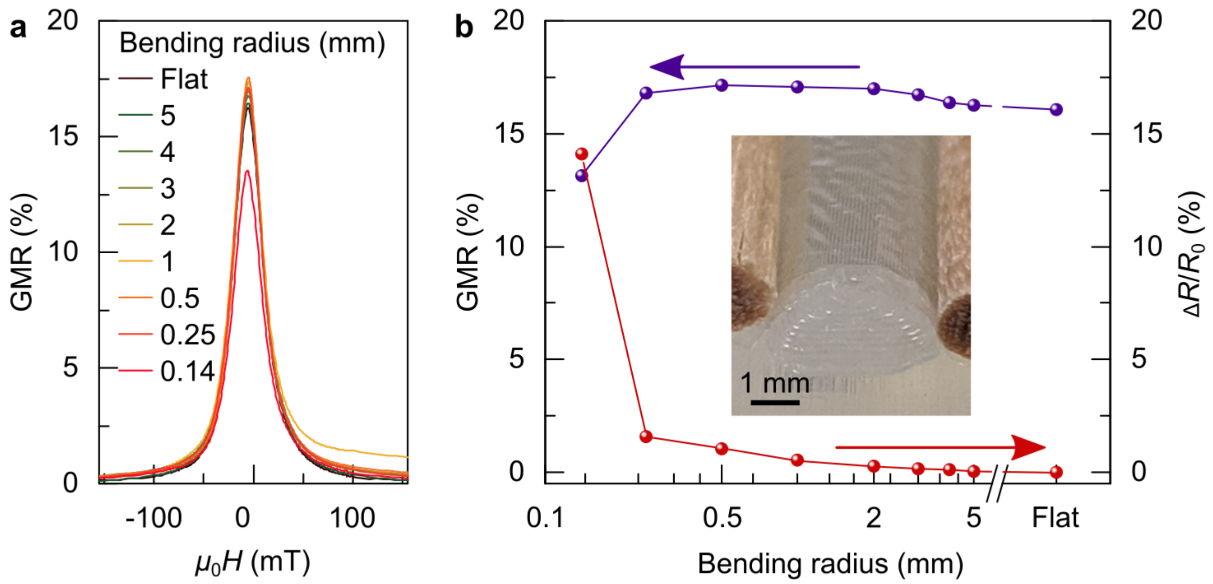

Supplementary Figure 16. Mechanical bending characterization of the GMR mesh sensor on a mylar foil. (a) The GMR curves measured of the mesh sensor bent to a different bending radius. (b) The GMR magnitude (left axis) and relative change of the base resistance ($(R_{bent} - R_{flat})/R_{flat}$) (right axis) over applied bending radii, inset shows GMR mesh sensor bent to 2 mm radius.

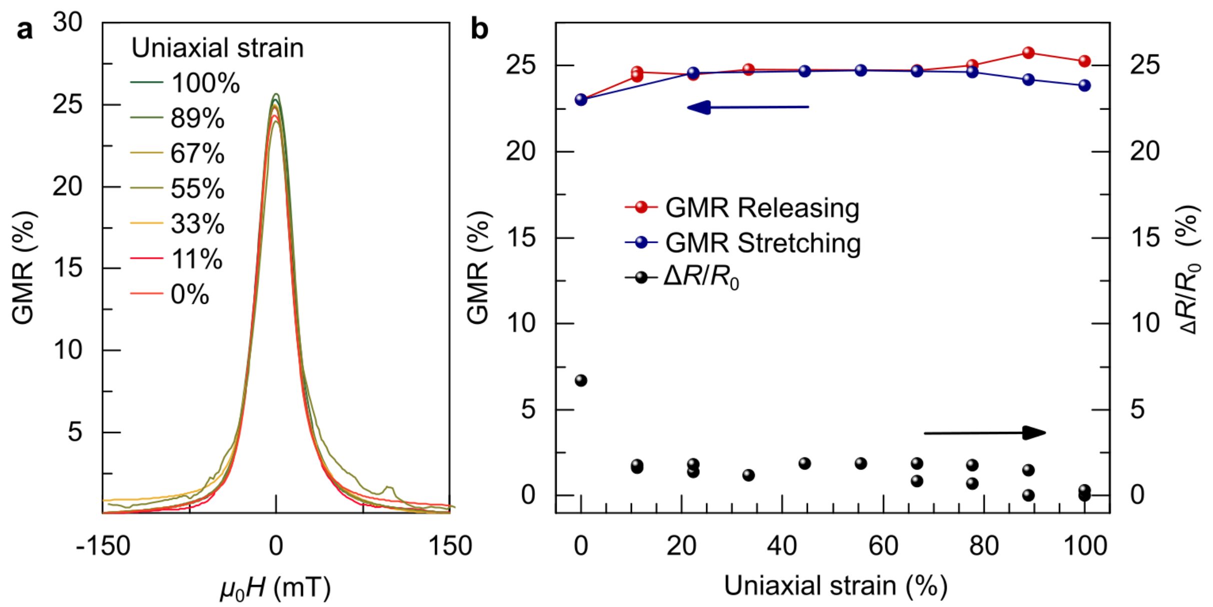

Supplementary Figure 17. Uniaxial stretching of the GMR mesh sensor. The GMR mesh sensor on a 3-μm-thick mylar foil is applied to a pre-stretched (500% linear elongation) Very High Bond (VHB) tape. The stack is released until the sensor lateral size is reduced twice (to 10 mm), this is taken as a reference 0 % strain state. The magnetoresistive response is measured under different applied tensile strain. (a) The GMR response of the mesh sensor under different applied tensile strains. (b) The magnitude of GMR response and variation of the base resistance as functions of applied uniaxial strain. Red and blue trend lines correspond to releasing and stretching of the sensor, completing a full cycle of deformation. Black points indicate the variation of the base resistance during the experiment.

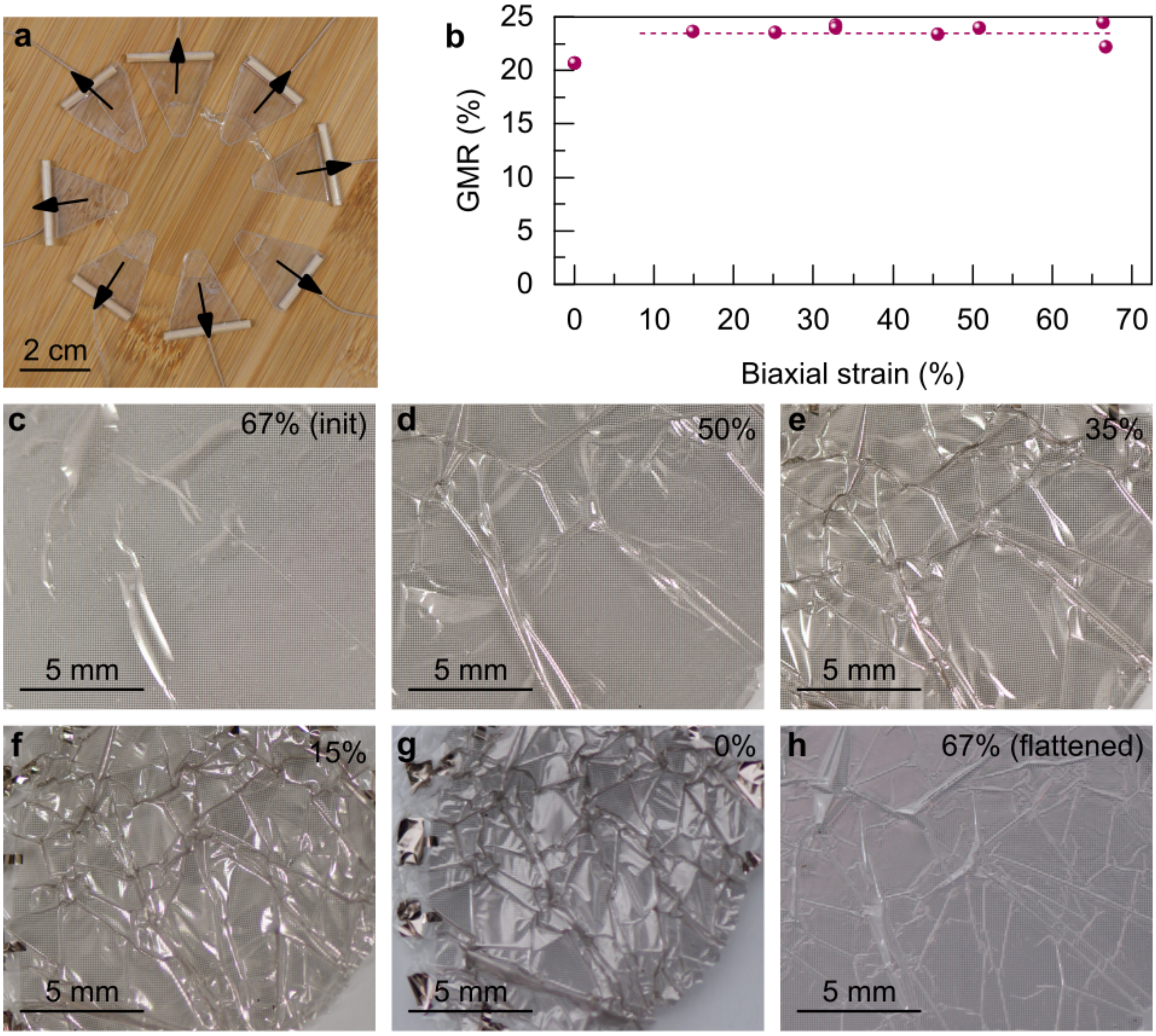

Supplementary Figure 18. Biaxial stretching of the GMR mesh sensor. (a) Photograph of biaxial stretching setup. A round shaped VHB tape is fixed around the perimeter with 8 evenly distributed clamps. Clamps are connected with a thread to a screw on a back side of a stage. By rotating the screw, uniform biaxial tension is applied to the VHB tape. A GMR mesh sensor with a lateral size of 20x20 mm^2 was adhered onto 100 % pre-stretched VHB tape. Magnitude of the applied strain was evaluated from variation of the sensor diagonal. The magnetoresistive response of sensor was evaluated by approaching the permanent magnet (350 mT on surface) to 5 mm distance. (b) The GMR magnitude measured over the applied biaxial strain. (c-h) A series of photographs showing the formation of wrinkles pattern during biaxial contraction of the sensor.

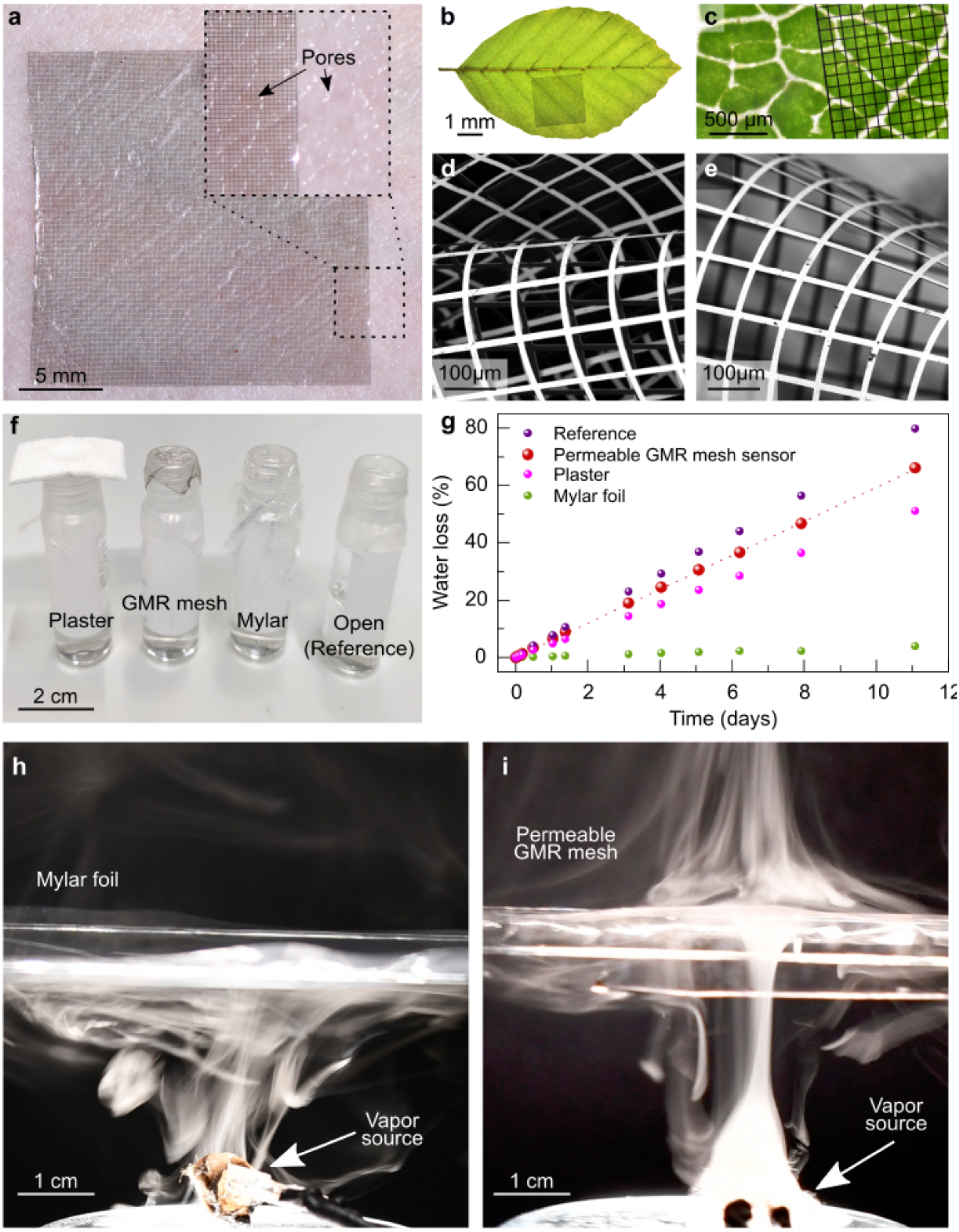

Supplementary Figure 19. Vapor permeable GMR membrane sensors. (a) GMR mesh sensor placed on skin. The sensor does not interfere the exposure of skin to light. (b-c) Optical photographs of perforated GMR mesh placed onto a leaf, suggesting low obstruction to the light and vapor flow. (d-e) SEM images of wrinkled perforated GMR mesh sensor. (f,g) Vapor permeability of the perforated GMR mesh sensor. (f) The experimental setup for estimating vapour permeability of the GMR mesh sensors. Flasks are filled with water and bottleneck is capped with the specified materials. Flasks are stored under fume hood at 35°C and water loss is

followed over time. (g) Amount of water evaporated from the flasks as a function of time. Data for permeable GMR mesh sensor with 10 µm linewidth and 100 µm pitch (19 % filling factor) is highlighted with dashed line. (h,i) Visualization of vapor flow through the permeable GMR mesh sensors. Reference mylar foil (h) and a permeable GMR mesh (i) are fixed above the vapour source, a cotton wool soaked with a mixture of propylene glycol and glycerine. The cotton is heated with a heater creating a flow of white color vapour. The reference mylar foil completely blocks the vapour flow. On the contrary, the permeable GMR mesh allows the vapor to pass through with a little scattering.

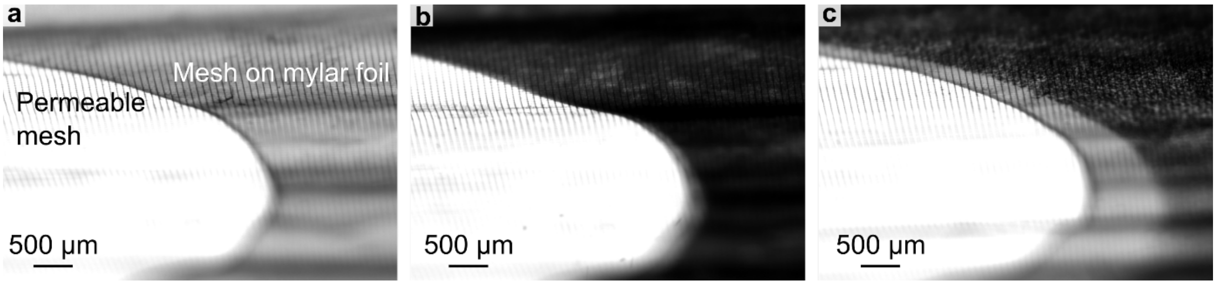

Supplementary Figure 20. Vapor permeability of the GMR mesh sensors. (a-c) A set of representative frames form the Supplementary Movie 4. (a) The GMR mesh sensor is positioned above the container. Left part of the mesh is perforated (brighter region) and the rest is left on continuous mylar foil (darker region). (b) The container is filled with warm water (about 60°C) and the water vapor flow is directed to the sensor mesh. The vapor flow passes unhindered through the perforated part of the sensor. At the same time the continuous mylar foil blocks the vapor flow and immediately covered with a condensated drops of water, which is causing darkening of this area. (c) After some time the vapor flow weakens and water drops evaporate from the surface of mylar foil, recovering the initial gray color of the foil. This process starts from the interface with a perforated part of the sensor, as gas flow there is the strongest, and further progresses over the mylar foil surface.

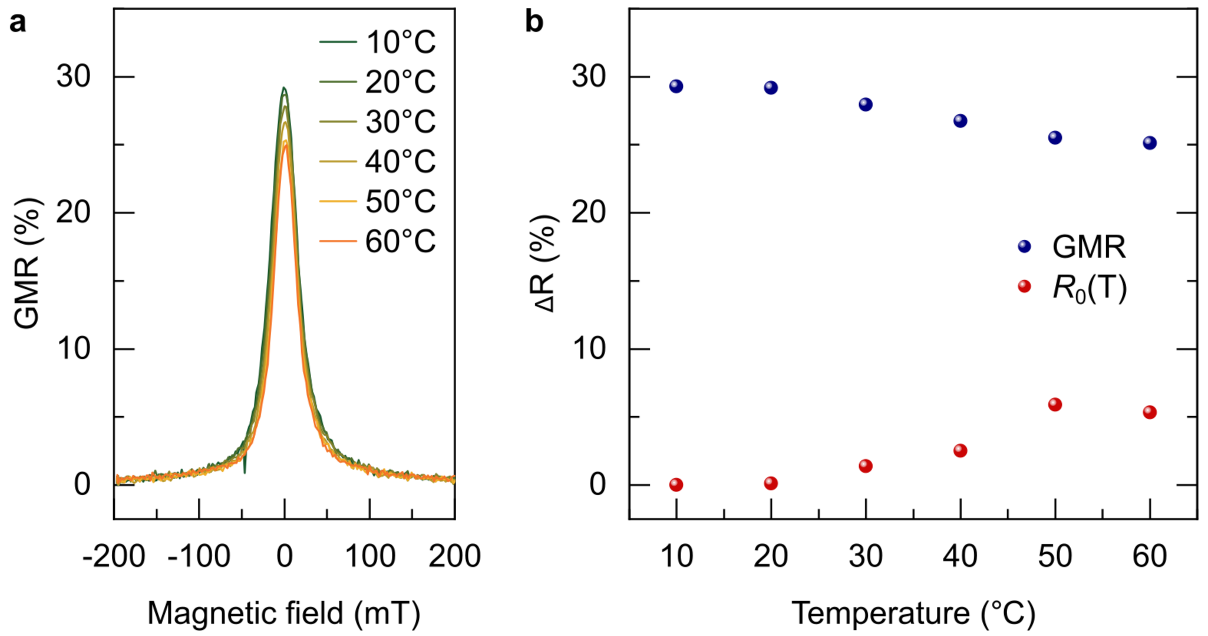

Supplementary Figure 21. Temperature stability of the transparent GMR mesh. The mesh sensor was placed onto a Peltier element and the device temperature was monitored using infrared camera. (a) The GMR curves measured of the mesh sensor at different temperatures. (b) The temperature variation of the base resistance (R_0) and GMR response of the sensor.

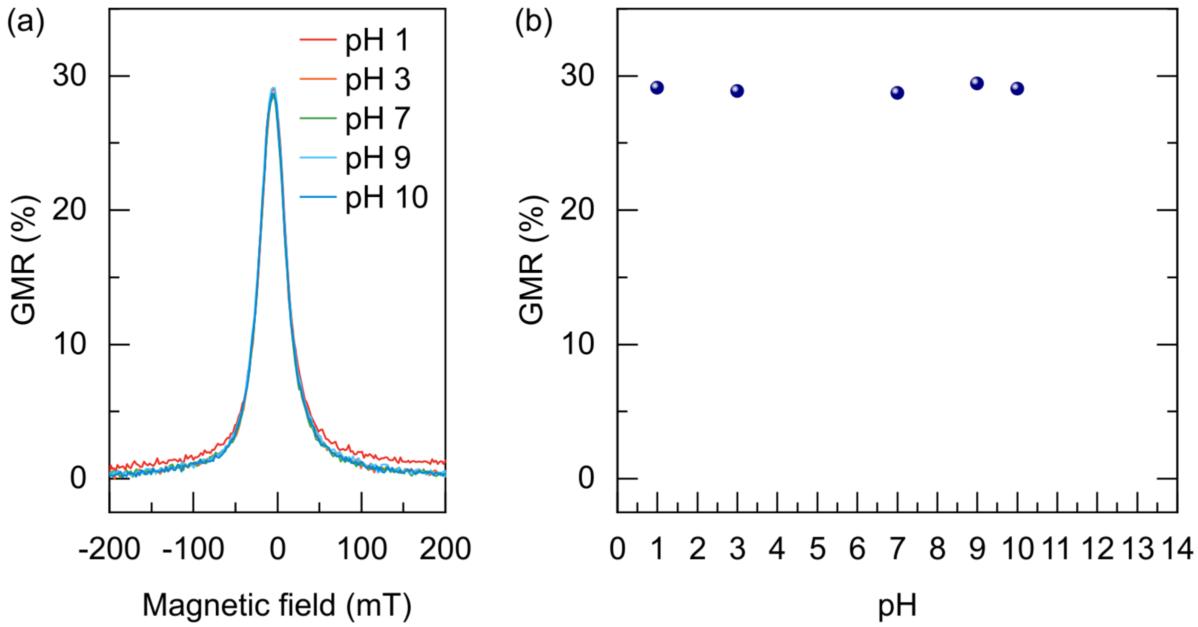

Supplementary Figure 22. The stability of the GMR mesh sensor upon immersion into media with different pH. (a) The GMR curves measured of the mesh sensors upon immersion into liquid media with different pH: citric acid (pH =1), vinegar (pH = 3), DI water (pH = 7), baking soda solution (pH = 9), soap solution (pH = 10). (b) The magnitude of GMR response of the encapsulated mesh sensor submerged into solutions with different pH.

Supplementary Note 1

Comparison of the transparent EMRT platform with an active transistor matrix

To compare the overall efficiency of our transparent electrical magnetoresistive tomography (EMRT) platform, we quantified its resolution, number of contacts, transmittance and energy consumption. In terms of number of contacts, the EMRT uses only 16 electrodes, which allows it to achieve a tracking resolution of about 1 mm (**Figure 2g of main text and Supplementary Figure 12**). Increasing the number of contacts improves the quality of the magnetic field profiles detected, but does not enhance the resolution guarantee[3]. Successful tracking requires only discriminating the maximum/minimum magnetic field value over the area influenced by the magnet. In this way, the platform could potentially use less electrodes (e.g., 8 electrodes) without sacrificing much in performance (**Supplementary Movie 3**). Achieving a similar resolution with standard approaches, would require an m x n active matrix of 70 x 70 transistors with an interelement separation of 1 mm, which amounts to 140, i.e., m + n, contacts, or about 10 times more than the EMRT. We note, however, that the precision per pixel and homogeneity of the active matrix would be superior, especially when measuring closer to the edges of the matrix. ERT is prone to artifacts when the detected objects approach the boundaries of the probed area[4,5]. A standard matrix of sensors without transistor–based switching would need as much as 4900 (m x n) contacts.

One obvious consequence of increasing the amount of contacts is a sharp decrease in transparency, as more traces need to be laid out on the design. For a mesh, this change can be quantified by the filling fraction (*f*), defined as the ratio W/G, where W and G are respectively the linewidth and spacing of the mesh[6,7]. Our transparent EMRT platform achieves a f = 19%, which corresponds to a transmittance T of 81%. Accounting the transmittance of the bare Mylar foil (92 %), the transmittance of the device will be defined as $T = T_{foil}*(1 - f)$ = 75%, which is consistent with our results (see Supplementary Figure 9). By contrast, an organic transistor matrix, for which we assume an active element (area covered by sensor + transistor) size of ~ 500 x 500 μm^2 (references[1,2]), trace width of 10 µm and mesh spacing of 1 mm would achieve an f of 510 µm / 1000 µm = 0.51 or a transmittance T of 45%. Nevertheless, the actual transmittance could be much lower as we are neglecting the contribution of the conductive traces going to and in between the unit cells. Furthermore, at a reading distance of 35 cm the transistor matrix would not be visually imperceptible, as the separation between active elements (~ 500 µm) would be larger than the grating acuity threshold for humans (~ 100 µm), and thus distinguishable for our eyes[7].

Regarding energy consumption, we contrast our EMRT platform with a state-of-the-art magnetosensitive active matrix[2]. A standard measurement with our EMRT system (16-contact configuration) uses about 1.8 mA, which multiplied by the contribution of the whole matrix of voltages measured (**Supplementary Table S1**) and the duration of sampling cycle of 0.9 ms, amounts to 114 mJ of electrical energy, that corresponds to power requirement of 570 µW. On the other hand, the energy consumed by the active matrix can be calculated from the bias current (50 µA) and the resistance of the magnetic sensors (22 kΩ), yielding 55 µW. Using this reference value and considering similar electronics as used for EMRT probing (0.9 ms per probing cycle), scaling it over a 70 x 70 matrix (4900 sensing units), would result in a total consumed

energy of 1267 mJ, which is almost 500 times larger than for the standard measurements with the EMRT platform. If we assume, however, that the sensors can be addressed with bias currents between 1-10 μA, typically used in other relevant works[8-11], for a current of 5 μA, the power consumed at each sensing unit would be 550 nW and the total power consumed is 2.6 mW. This value is still almost 5 times larger than the power consumed by the EMRT platform in standard mode.

Supplementary Tables:

Supplementary Table S1. Matrix of voltages (in mV) during an EMRT measurement with a 1.8 mA bias current. The rows and columns represent all possible combinations of contacts for data acquisition performed in adjascent-adjascent configuration. Values of 0 indicate the contacts occupied already by the current sourcing electrodes during each measurement cycle.

	1-2	2-3	3-4	4-5	5-6	6-7	7-8	8-9	9-10	10-11	11-12	12-13	13-14	14-15	15-16	16-1
1-2	0	0	-1.175	-0.2	-0.577	-0.582	-0.324	-0.077	-0.268	-0.473	-0.406	-0.137	-0.653	-2.085	-7.151	0
2-3	0	0	0	-0.933	-1.971	-1.355	-0.588	-0.134	-0.411	-0.623	-0.463	-0.147	-0.608	-1.347	-1.838	-0.921
3-4	-1.178	0	0	0	-6.93	-1.884	-0.558	-0.107	-0.315	-0.414	-0.266	-0.073	-0.296	-0.529	-0.514	-0.193
4-5	-0.213	-0.938	0	0	0	-0.897	-0.209	-0.048	-0.11	-0.131	-0.084	-0.032	-0.086	-0.133	-0.117	-0.05
5-6	-0.577	-1.969	-6.93	0	0	0	-1.232	-0.199	-0.503	-0.545	-0.302	-0.08	-0.289	-0.435	-0.347	-0.114
6-7	-0.596	-1.365	-1.894	-0.882	0	0	0	-0.956	-1.83	-1.423	-0.641	-0.166	-0.521	-0.665	-0.45	-0.142
7-8	-0.331	-0.592	-0.564	-0.194	-1.239	0	0	0	-7.766	-2.373	-0.734	-0.165	-0.482	-0.525	-0.301	-0.086
8-9	-0.076	-0.125	-0.106	-0.03	-0.194	-0.939	0	0	0	-1.188	-0.262	-0.05	-0.147	-0.145	-0.075	-0.016
9-10	-0.281	-0.414	-0.324	-0.103	-0.513	-1.835	-7.771	0	0	0	-1.42	-0.263	-0.665	-0.592	-0.294	-0.082
10-11	-0.471	-0.613	-0.406	-0.108	-0.544	-1.416	-2.364	-1.176	0	0	0	-1.15	-2.242	-1.435	-0.576	-0.133
11-12	-0.418	-0.47	-0.278	-0.077	-0.312	-0.645	-0.739	-0.271	-1.421	0	0	0	-7.913	-2.067	-0.609	-0.141
12-13	-0.14	-0.144	-0.075	-0.016	-0.081	-0.157	-0.158	-0.048	-0.251	-1.152	0	0	0	-1.075	-0.233	-0.044
13-14	-0.668	-0.612	-0.309	-0.084	-0.302	-0.525	-0.489	-0.161	-0.668	-2.264	-7.918	0	0	0	-1.346	-0.252
14-15	-2.085	-1.336	-0.528	-0.119	-0.438	-0.659	-0.519	-0.147	-0.585	-1.448	-2.061	-1.066	0	0	0	-1.046
15-16	-7.168	-1.845	-0.525	-0.113	-0.359	-0.456	-0.307	-0.084	-0.297	-0.596	-0.611	-0.239	-1.343	0	0	0
16-1	0	-0.928	-0.204	-0.044	-0.126	-0.144	-0.09	-0.026	-0.081	-0.149	-0.137	-0.052	-0.248	-1.056	0	0

Supplementary Table S2. Comparison of parameters between different active transistor matrices and our EMRT platform over an area of 70 mm x 70 mm. For references[2,11], the theoretical transmittance values are calculated assuming an active element size of 500 μm x 500 μm as described in the section above. For reference[9], the mesh-like configuration presented in their paper is used. The estimated energy per frame is obtained from the standard operating currents and the bias voltage or sensor resistance if available, multiplied by the total number of transistors (4900) needed to tile the 70 mm x 70 mm area. The sampling and switching rates of the external electronics was assumed to similar to the ones used for our EMRT platform.

**Reference**	**Matrix type**	**Transmittance (%)**	**Estimated energy per frame (mJ)**	**Number of contacts for 70 x 70 mm² area**
12	Pressure sensors with organic active transistors	73 (theoretical)	461	140
13		75 (theoretical)	1385	140
14		90 (theoretical)	3462	140
15	Magnetic sensors with organic active transistors	45 (theoretical)	1267	140
16		-	16170.00	140
**This work**	**Magnetoreceptive tomography**	**75**	**0.57**	**16**
17	Graphene transistors	80	20.78	140
18		>90	277	140
19	Carbon Nanotubes Transistors	70	346.3	140
20	Pentace transistors	45 (theoretical)	462	140

References

1. Borchert, J. W. *et al.* Flexible low-voltage high-frequency organic thin-film transistors. *Sci. Adv.* **6**, eaaz5156 (2020).
2. Kondo, M. *et al.* Imperceptible magnetic sensor matrix system integrated with organic driver and amplifier circuits. *Sci. Adv.* **6**, eaay6094 (2020).
3. Harrach, B. & Ullrich, M. Resolution Guarantees in Electrical Impedance Tomography. *IEEE Trans. Med. Imaging* **34**, 1513–1521 (2015).
4. Borsoi, R. A., Aya, J. C. C., Costa, G. H. & Bermudez, J. C. M. Super-resolution reconstruction of electrical impedance tomography images. *Comput. Electr. Eng.* **69**, 1–13 (2018).
5. Boyle, A., Adler, A. & Lionheart, W. R. B. Shape Deformation in Two-Dimensional Electrical Impedance Tomography. *IEEE Trans. Med. Imaging* **31**, 2185–2193 (2012).
6. Ghosh, D. S., Chen, T. L. & Pruneri, V. High figure-of-merit ultrathin metal transparent electrodes incorporating a conductive grid. *Appl. Phys. Lett.* **96**, 041109 (2010).
7. Pan, C. *et al.* Visually Imperceptible Liquid-Metal Circuits for Transparent, Stretchable Electronics with Direct Laser Writing. *Adv. Mater.* **30**, 1706937 (2018).
8. Someya, T. *et al.* A large-area, flexible pressure sensor matrix with organic field-effect transistors for artificial skin applications. *Proc. Natl. Acad. Sci. USA* **101**, 9966–9970 (2004).
9. Someya, T. *et al.* Conformable, flexible, large-area networks of pressure and thermal sensors with organic transistor active matrixes. *Proc. Natl. Acad. Sci. USA* **102**, 12321–12325 (2005).
10. Sun, Q. *et al.* Transparent, Low-Power Pressure Sensor Matrix Based on Coplanar-Gate Graphene Transistors. *Adv. Mater.* **26**, 4735–4740 (2014).

11. Ren, X. *et al.* A Low-Operating-Power and Flexible Active-Matrix Organic-Transistor Temperature-Sensor Array. *Adv. Mater.* **28**, 4832–4838 (2016).
12. Someya, T., *et al.* Conformable, flexible, large-area networks of pressure and thermal sensors with organic transistor active matrixes. *Proc. Natl. Acad. Sci. USA* **102**, 12321–12325 (2005).
13. Zang, Y., *et al*. Flexible suspended gate organic thin-film transistors for ultra-sensitive pressure detection. *Nat. Commun.* **6**, 6269 (2015).
14. Liang, J., Tong K., & Pei Q. *Adv. Mater.* **28**, 5986-5996 (2016).
15. Kondo, M., *et al*. Imperceptible magnetic sensor matrix system integrated with organic driver and amplifier circuits. *Sci. Adv.* **6**, eaay6094 (2020).
16. Becker, C., et al. A new dimension for magnetosensitive e-skins: active matrix integrated micro-origami sensor arrays. *Nat. Commun.* **13**, 2121 (2022).
17. Sun, Q., *et al.* Transparent, Low-Power Pressure Sensor Matrix Based on Coplanar-Gate Graphene Transistors. *Adv. Mater.* **26**, 4735-4740 (2014).
18. Lee, S., *et al.* A transparent bending-insensitive pressure sensor. Nature Nanotech. **11**, 472–478 (2016).
19. Nela, L., *et al.* Large-Area High-Performance Flexible Pressure Sensor with Carbon Nanotube Active Matrix for Electronic Skin. *Nano Lett.* **18**, 2054–2059 (2018).
20. Ren, X., *et al*. A Low-Operating-Power and Flexible Active-Matrix Organic-Transistor Temperature-Sensor Array. *Adv. Mater.* **28**, 4832-4838 (2016).
