# Supplementary material for: Scalable magnetoreceptive e-skin for energy-efficient high-resolution interaction towards undisturbed extended reality
Source: Nat Commun. 2025 Feb 14;16:1647. doi: 10.1038/s41467-025-56805-x (PMC11828903; doi:10.1038/s41467-025-56805-x)
Supplement: Supplementary file 2 — Description of Additional Supplementary Information [file 41467_2025_56805_MOESM2_ESM.docx]

**Description of Additional Supplementary Files**

File Name: Supplementary Movie 1

Description: Interactive electronic skins as pattern recognition devices based on large-area magnetic field mapping. The permeable large-area magnetoreceptor (size: 70x70 mm^2^) is placed onto a wrist of a person and is used to unlock a smartphone via input of 4-digid pattern PIN code in a contactless manner. Resistance state of the sensor is monitored using EMRT algorithm. Nine markers are displayed on the smartphone screen and are used to input pattern PIN code. Magnetic stylus is used as an input device. As the stylus is approached and hovered above the mesh sensor its trajectory is recorded and transferred to the smartphone. First, wrong 4-digid PIN code was entered and “Wrong Pin” message is displayed. Stylus is removed from the mesh sensor to reinitiate input of the PIN code. As the correct PIN code inserted “Pin OK” message displayed.

File Name: Supplementary Movie 2

Description: Transparent GMR mesh sensor as a touchless human-machine interface for handwriting on light-emitting surfaces. The optically imperceptible GMR sensor (lateral size: 70x70 mm^2^) is mounted on a corner of a computer screen, its outline is indicated by white dashed line. The sensor is positioned metallic layers towards the screen, indicating possibility of touchless interactivity. The resistance state of the sensor is monitored using the EMRT algorithm. This setup allows reconstructing the resistance map over the sensor area and motion-tracking of complex trajectories of local magnetic stimuli. In this example, the large-area magnetoreceptor is used for input of handwritten word “Hello”. As the mesh sensor is approached by the stylus, the spatial position of the resistance drop due to a localized magnetic stimuli is detected and its apex is displayed by an orange point in “Reconstruction view” window. To write a character “H” the magnetic stylus is hovered over the mesh sensor, its trajectory is recorded and displayed in “Trajectory tracking” window. When the letter is finished, the stylus is removed from the mesh sensor and the software is programmed for an input of a new character. This operation is repeated until complete word “Hello” is written.

File Name: Supplementary Movie 3

Description: Magnetic field mapping using the EMRT algorithm in 8-contacts geometry. A reference GMR mesh sensor (40x40 mm^2^ size) is prepared on Si/SiO_x_ substrate and its resistance state is monitored relying on EMRT with 8-contacts readout geometry. In spite of the reduction in the number of contacts, the mesh sensor reliably maps local magnetic stimuli coming from the magnetic stylus.

File Name: Supplementary Movie 4

Description: Water vapor permeability of a perforated GMR mesh sensor. A partially perforated GMR membrane sensor is located above the container. Camera is oriented with incidence angle of roughly 10 degrees to the sensor plane. This allows follow the formation of water drops on the sensor. During the experiment, the container is filled with the warm water (60°С) and the vapor flow is directed towards the sensor. Vapor passes unhinderedly through the breathable sensor 2 membrane (left bright part). In contrast, continuous polymer foil (gray part) hinders vapor flow. This is followed with intensive condensation of water on the sensor surface, represented with darkening of the continuous part of the sensor. After some time the sensor starts slowly drying, which is indicated by moving of gray contrast starting from the perforated part.

File Name: Supplementary Movie 5

Description: Fine motion gesture recognition for virtual reality applications. The vapour-permeable GMR sensor (size: 55x55 mm^2^) is placed onto a forearm of a person and used as an on-skin input device for virtual reality (VR). The sensor is used as an on-skin interface for manipulating virtual objects. In this example, a 3D model of a protein molecule is biaxially rotated in virtual reality environment driven by Unity 3D platform. Monitor on the background reproduces field of view of a person wearing an Oculus Quest 2 VR headset. The magnetoresistive response of the GMR mesh sensor is monitored relying on EMRT algorithm, enabling reconstruction of local resistance change over the large-area magnetoreceptor. An inset in bottom-right corner of a frame indicates local drop of the resistance by a dark orange spot. Rotation vector of a protein molecule is calculated from the position of magnetic input stimuli. A reference point is positioned in the center of a sensor area, this was assigned as (x;y) = (0;0). To enable interaction, a permanent magnet was affixed onto the index finger of a person. As the sensor element is approached with a magnet, its relative position (in the form of x and y coordinates) is recorded and transformed into angular velocities λ and µ, corresponding to azimuthal and polar rotation angles Θ and Ψ. Angular velocities of rotary motion of the protein is assigned to zero as the magnetic stimuli is removed from the sensor element.

File Name: Supplementary Movie 6

Description: Multipoint interaction using soft magnetic skins. Two mechanically flexible magnetic skins consisting of a composite of NdFeB-PDMS (7:3 mass ratio) that provide about 20 mT magnetic stray field at the surface are adhered onto a user’s fingertips and used to interact with large-area magnetoreceptor (size: 70x70 mm^2^). The EMRT readout algorithm applied to the magnetoreceptor is able to recognize if the magnetoreceptor is approached with one or two magnetic objects. A single point of interaction can be seen as a symmetric spot at the reconstructed resistance map. If two magnetic stimuli triggered simultaneously, two peaks can be observed on the reconstructed image. If the two magnets are closely positioned, the GMR response reconstructed by the EMRT algorithm appears as a singular spot of a non-circular (i.e., elongated) shape. In this case, the two interaction points can be separated by a straightforward numerical analysis of the peak deconvolution. In this example, the measured data are analysed using double-Gaussian fit shown in the “Numerical fit” plot. The position of each peak corresponds to the reconstructed position of the localized magnetic stimulus. When the large-area magnetoreceptor is approached and pressed by bare fingers (without the magnetic skin), the EMRT algorithm returns no input signal, as the sensor is sensitive to magnetic stimuli only. The magnetoreceptor is covered by a 125-µm-thick PET foil to prevent electrical shortcut to the fingers.

File Name: Supplementary Movie 7

Description: Multipoint interaction using permanent magnets. A large-area transparent magnetoreceptor (size: 120x120 mm^2^) is placed on top of a steel plate and is used to locate up to four cube-shaped permanent magnets. The magnets are sequentially placed within the area of the magnetoreceptor and kept in place by the steel plate not allowing them to collide. The auto-scaled reconstructed resistance maps are shown live on a computer monitor. The visualization is done simultaneously using Matlab software (rainbow colorcode, right panel) and in Wolfram Mathematica (orange tones colorcode, left panel). The time delay between the two panels is related to the data upload time. The local change in the resistance induced by the approached individual magnets is represented as blue (violet) spots on the reconstructed resistance maps. The positions of the four magnets are recognized on the EMRT reconstructed resistance maps.

File Name: Supplementary Movie 8

Description: Magnetoreceptive contact lens for augmented reality applications. Adhering the imperceptible magnetoreceptive membrane to a contact lens turns the typically passive surface of the lens into an active device for augmented reality, which can track motion using the EMRT algorithm. In this demonstration, the magnetoreceptive contact lens performs two operations: (i) Taking a snapshot of an object of interest and (ii) Operating digital magnification of a taken snapshot. The software is programmed to select the operating mode (“Take a snapshot” and “Magnify”) depending on the intensity of the input signal, which is proportional to the distance between a magnetoreceptive contact lens and the input magnetic object. (i) To take a snapshot of an object of interest, a selection marker is dragged on it from top-right corner of the field of view. Selection of a snapshot is confirmed when signal intensity crosses the corresponding threshold. (ii) The magnification of a selected snapshot is specified in a range from 1x to 5x depending on the position of a magnetic stimuli along the circumference of the lens.
